# Selective Chemical Conversion of Sugars in Aqueous Solutions without Alkali to Lactic Acid Over a Zn-Sn-Beta Lewis Acid-Base Catalyst

**DOI:** 10.1038/srep26713

**Published:** 2016-05-25

**Authors:** Wenjie Dong, Zheng Shen, Boyu Peng, Minyan Gu, Xuefei Zhou, Bo Xiang, Yalei Zhang

**Affiliations:** 1 State Key Laboratory of Pollution Control and Resources Reuse, National Engineering Research Center of Facilities Agriculture, Key Laboratory of Yangtze River Water Environment of Ministry of Education, College of Environmental Science and Engineering, Tongji University, Shanghai 200092, China; 2 Department of Chemistry, Tongji University, Shanghai 200092, China

## Abstract

Lactic acid is an important platform molecule in the synthesis of a wide range of chemicals. However, in aqueous solutions without alkali, its efficient preparation via the direct catalysis of sugars is hindered by a side dehydration reaction to 5-hydroxymethylfurfural due to Brønsted acid, which originates from organic acids. Herein, we report that a previously unappreciated combination of common two metal mixed catalyst (Zn-Sn-Beta) prepared via solid-state ion exchange synergistically promoted this reaction. In water without a base, a conversion exceeding 99% for sucrose with a lactic acid yield of 54% was achieved within 2 hours at 190 °C under ambient air pressure. Studies of the acid and base properties of the Zn-Sn-Beta zeolite suggest that the introduction of Zn into the Sn-Beta zeolite sequentially enhanced both the Lewis acid and base sites, and the base sites inhibited a series of side reactions related to fructose dehydration to 5-hydroxymethylfurfural and its subsequent decomposition.

In general, lactic acid is a key chemical platform molecule for the synthesis of a wide range of chemicals, such as pyruvic or acrylic acid^1^,^2^. Currently, lactic acid has emerged as a renewable building-block chemical in a new generation of materials including biodegradable plastics or solvents^3^,^4^. The industrial production of lactic acid typically involves anaerobic fermentation in aqueous solutions of saccharides or direct chemical catalytic transformations. The former route operates under moderate conditions and sequentially overcomes the major obstacle of the thermal instability of the biomass. However, some disadvantages exist including a limited space-time yield and the coproduction of large amount of wastes due to the addition of calcium carbonate and sulphuric acid for neutralization.

As a substitute, lactic acid can be synthesized from mono- or polysaccharide by chemical catalytic routes using homogeneous^5^,^6^,^7^,^8^ or heterogeneous catalysts^9^,^10^,^11^,^12^,^13^,^14^,^15^,^16^. Catalytic processes may be scalable with the development of improved process design options, which would result in higher productivity and reduce costs related to product work-up. Heterogeneous catalysts are advantageous from a separation efficiency standpoint because they can be easily recovered by filtration in contrast to homogeneous ones. Among of the multiple heterogeneous catalysts in this area, Taarning *et al.* in 2010 attracted much attention, and reported that the Sn-Beta zeolite, which acts as a Lewis acid, efficiently catalyzed the transformation of saccharides into methyl lactate in a methanol system at 160 °C for 20 hours, and the yields of methyl lactate as high as 64% from glucose. However, the yield of lactic acid was only approximately 27% in aqueous solutions under the same conditions^10^. Because the lactic acid and other organic acids, which is a Brønsted acid catalyst that competes with the original Sn-Beta Lewis acid, worked irreversibly for the direct formation of 5-hydroxymethylfurfural (HMF) and its decomposition products from saccharides. Due to the unfavourable effect of these Brønsted acids, many researchers have developed base-catalyzed aqueous solution systems, such as Ba(OH)_2_^17^ and polymer catalysts in a NaOH solution^18^. However, a stoichiometric amount of base is required to obtain a high lactate yield, followed by recovery of lactic acid using the same stoichimetric amount of acid for neutralization. In aqueous solutions without a base, a high lactic acid yield (approximately 50%) with a satisfactory rate remains a challenge, because the produced lactic acid acts as a Brønsted acid catalyst to further catalyze fructose to HMF and its derivatives.

Herein, we report that a previously unappreciated combination of common two metal mixed catalysts (Zn-Sn-Beta zeolite) synergistically promoted this reaction. In an aqueous solution without a base, conversions exceeding 99% of the sucrose and with a lactic acid yield of 54% were achieved within 2 hours at 190 °C under ambient air pressure. A preliminary study of the acid and base properties of the Zn-Sn-Beta zeolite indicated that the introduction of Zn into the Sn-Beta zeolite enhanced both Lewis acid and base sites, which further inhibits a series of side reactions related to fructose dehydration to HMF and its subsequent decomposition. This route has great potential for industrial application.

## Results and Discussion

### Catalyst preparation and characterization

The zeolite catalysts were synthesized using a more convenient and scalable method known as solid-state ion exchange (SSIE) rather than by a complicated and small-scale hydrothermal approach to avoid longer crystallization times and higher environmental risks due to the use of hydrofluoric acid as a mineralizing agent^19^. To explore the possible structural differences among the zeolite catalysts, XRD patterns were collected (Fig. S1). All of the zeolites exhibited well-defined reflections with a BEA topology. The similar patterns of Beta and deAl-Beta suggest that the dealumination process does not significantly affect the textural properties of the BEA zeolite. After the introduction of Zn and Sn, Zn-Sn-Beta only exhibited the characteristic diffraction peaks of the BEA topology and did not exhibit any reflection peaks associated with any crystalline metal oxides, such as SnO_2_ and ZnO. This result indicates that crystalline metal oxides were not formed, and Zn and Sn were successfully incorporated into the framework of the Beta zeolite. However, the existence of amorphous metal oxide clusters cannot be excluded based on the XRD data^20^. A matrix contraction/expansion of the zeolite structure could be deduced by the position of the diffraction peak (302) at 2*θ* = 22.5–22.6°^21^,^22^. The *d*_302_ spacing decreased from 3.968 (Beta, 2*θ* = 22.382°) to 3.931 Å (deAl-Beta, 2*θ* = 22.598°), suggesting matrix contraction due to the dealumination process. In contrast, a matrix expansion of the zeolite structure occurred after the introduction of Zn and Sn based on the increase in the *d*_302_ spacing from 3.931 (deAl-Beta, 2*θ* = 22.598°) to 3.948 (Zn-Beta, 2*θ* = 22.498°), 3.951 Å (Sn-Beta, 2*θ* = 22.484°) and 3.945 Å (Zn-Sn-Beta, 2*θ* = 22.516°). This expansion in the BEA framework also confirmed the successful incorporation of Zn and Sn into the framework of the deAl-Beta zeolite^23^. Raman spectroscopy can detect very small amounts of transition metals in zeolites. Figure S2 shows the Raman spectra of ZnO, SnO_2_ and Zn-Sn-Beta. It is important to note that no ZnO or SnO_2_ were observed in the Raman spectrum of Zn-Sn-Beta. This result indicates that the Zn-Sn-Beta zeolite does not appear to have extra-framework ZnO and SnO_2_ which is consistent to the XRD data. In addition, Hermans *et al.* confirmed that the presence of framework Sn in the Sn-Beta zeolite prepared by SSIE using dynamic nuclear polarization surface enhanced nuclear magnetic resonance^24^.

Table S1 summarizes the physicochemical properties of the zeolites. The samples exhibited similar BET surface areas (581–610 m^2^g^−1^), total volumes (0.348–0.374 mlg^−1^) and micropore volumes (0.184–0.191 mlg^−1^). The Si/Al ratio sharply increased from 25 for Beta to more than 1700 for deAl-Beta, indicating that Al was completely removed by the nitric acid treatment. Using ICP analysis, the quantity of Zn or Sn in the samples was identical to the desired value, which confirms that SSIE is an efficient method. The nitrogen physisorption isotherms of the samples presented belong to type I (microporous materials) with a narrow hysteresis loop located at 0.5 < P/P_0_ < 0.8, which may be due to the presence of interparticle voids or a random distribution of mesopores in the structure (Fig. S3a). The mesoscopic pores are approximately 3.69 nm based on the pore size distribution determined from the desorption branch using the BJH method (Fig. S3b).

### Conversion of sugars to lactic acid in an aqueous solution without alkali

The materials were tested as catalysts for the conversion of sugars to lactic acid in aqueous solutions. Table 1 lists the results of glucose transformation to lactic acid. The conversion of glucose increased to 78% from 21% using the rude Beta zeolite as a catalyst. However, both yields of lactic acid are negligible (Table 1, entries 1 and 2). The main products consisted of HMF, in a yield of 14.6% and its derivatives (i.e., 2,5-furandicarboxaldehyde, furfural, 2-methyl furan, etc.) with the yield of 7.5%. The catalytic activity of the rude Beta zeolite can be ascribed to its internal Brønsted and Lewis acids. When the dealuminated Beta zeolite (de-Al Beta) was used, the conversion of glucose decreased to 37% and the yield of lactic acid was still very low (entry 3). In the presence of Sn-Beta or Zn-Beta zeolite, the glucose was nearly completely converted, and the yield of lactic acid increased to 23% and 17%, respectively, along with larger amounts of HMF and its derivatives (entries 4 and 5). The Lewis acid is capable of catalysing glucose to fructose via an isomerization reaction^25^ followed by subsequent reactions that produce lactic acid^3^. In the case of SnO_2_ and ZnO, they give rise to low yields of lactic acid (entries 7 and 8).

**Figure 1:**
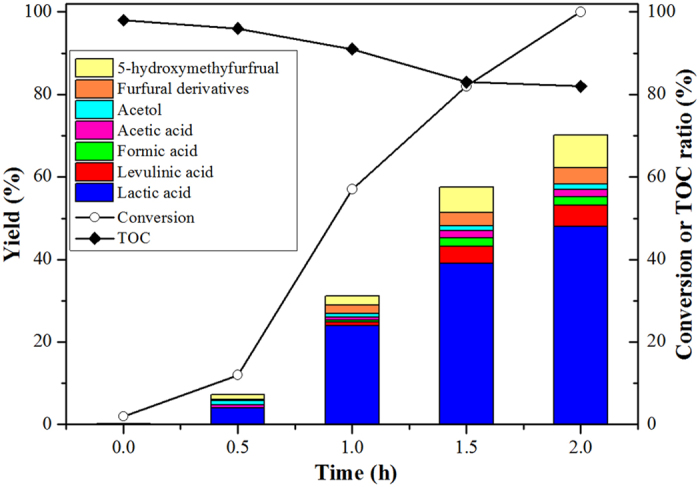
Product distribution over the Zn-Sn-Beta zeolite under different reaction times (225 mg glucose, 10 ml water, 160 mg catalyst, 190 °C).

When the Zn-Sn-Beta zeolite, which contains both Zn and Sn, was used, the reaction proceeded with a surprising lactic acid yield of 48% and complete conversion of glucose at 190 °C for 2 hours (entry 6). We further examined the glucose conversion and product distributions using the Zn-Sn-Beta zeolite as a function of reaction time (Fig. 1). As the reaction time increased from 0.5 to 2.0 hours, the conversion of glucose increased from 12 to 100%, the yield of lactic acid increased from 4 to 48%, and the total organic carbon (TOC) relatively decreased from 96 to 82%. For a longer reaction time, the yield of lactic acid does not further change (Fig. S4a). This result indicates that 2 hours is sufficient for the catalytic reaction, and lactic acid is stable in the system. Furthermore, the yield of lactic acid was nearly unchanged (i.e., approximately 24%) when the Zn-Sn-Beta was removed by hot-filtration after 1 hour (Fig. S4b). In this case, the catalytic activity for the production of lactic acid was due to the Zn-Sn-Beta zeolite. In three types of gas environments (i.e., air, nitrogen and oxygen), the yields of lactic acid remained nearly the same under ambient pressure (Fig. S5). This result is in contrast to previous reports that emphasized the fact that lactic acid is produced under a high pressure inert atmosphere^4^,^26^, and therefore, the ambient air pressure is an advantage for industrial applications of this catalyst in the future. The recycling ability of the Zn-Sn-Beta zeolite for the conversion of glucose was investigated, and the results are shown in Fig. S6. A decrease in the yield of lactic acid was observed within five cycles. The leaching of metals during the reaction may be the cause. In general, Zn-Sn-Beta is a potential catalyst for future application in the conversion of biomass to lactic acid. However, a more stable catalyst is required.

The conversation of different hexoses (i.e., glucose, fructose, galactose and mannose) and disaccharides (i.e., sucrose, lactose and cellobiose) via the Zn-Sn-Beta zeolite was also investigated. Simultaneously changing the solvent from water to methanol leads to the formation of the corresponding free lactic acid to methyl lactate (Table S2). More than 99% conversion of the substrates was observed for the entire experimental group. For hexoses, considerable yields of lactic acid (45–52%) or methyl lactate (25–30%) were obtained (entries 1 to 5). Sucrose gives a slightly higher yield of lactic acid (54%) and methyl lactate (32%) than that from the hexoses (entry 6), which is consistent with previous results^10^,^12^. In contrast to sucrose, the yields of lactic acid and methyl lactate were relatively low using lactose and cellobiose as the substrates (entries 7 to 8). Interestingly, the yields of lactic acid in aqueous solutions were higher than those of methyl lactate in methanol solutions with the Zn-Sn-Beta zeolite compared to other studies^9^,^10^,^13^. The reason is unclear and needs to be further investigated in detail.

### Analysis of acid and base properties

**Figure 2:**
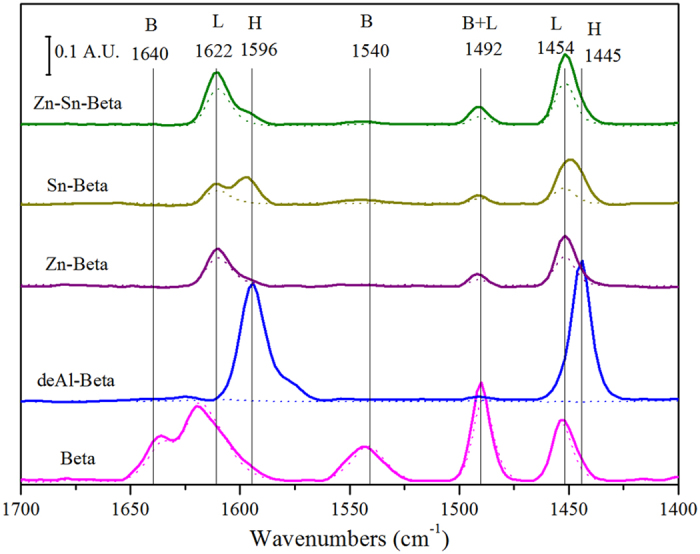
FT-IR spectra of pyridine adsorbed at 150 °C (solid line) and 250 °C (dotted line) over different Beta zeolites.

The excellent catalytic activity of the Zn-Sn-Beta zeolite may be explained by its acid properties, which investigated via pyridine adsorption on the catalyst surface using FT-IR spectroscopy. Figure 2 shows the infrared spectra of pyridine adsorbed on the Beta zeolites at 150 °C (solid line) and 250 °C (dotted line). After the dealumination of the Beta zeolite by the HNO_3_ solution, all of the bands corresponding to the Lewis acid sites (1454 and 1622 cm^−1^, bands labelled L in Fig. 2) and Brønsted acid sites (1540 and 1640 cm^−1^, bands labelled B in Fig. 2) disappeared, and only intense bands located at 1445 and 1596 cm^−1^ (bands denoted as H) were obtained for the de-Al Beta zeolite and correspond to hydrogen-bonds between pyridine and the surface silanol groups of zeolite^27^,^28^,^29^. Due to the weak interactions, these bands disappear when the thermal treatment was increased from 150 °C to 250 °C. The presence of a stronger Lewis acid rather than a Brønsted acid in Zn-Sn-Beta than Zn-, Sn-beta zeolites was demonstrated by the bands assigned to pyridine. The generation of Lewis acidity was due to the incorporation of Sn or Zn in a tetrahedral coordination within the silica framework. An extra band corresponding to the vibration of pyridine associated with both Lewis and Brønsted acid sites was observed at 1492 cm^−1^ (band labelled B+L in Fig. 2). Table S3 summarizes the quantity of Brønsted acid sites and Lewis acid sites in the catalysts over different temperatures. Although weakening gradually as the thermal treatment temperature increased, these bands due to the Lewis acid sites in Zn-Sn-Beta were observed in the spectra recorded at 150 to 450 °C, indicating that these Lewis acid sites have a strong characteristic. The maximum quantity of Lewis acid sites was 0.17 mmolg^−1^ at 150 °C in the bimetallic-loaded Zn-Sn-Beta zeolite, which is agreement with the requirements of an ideal catalyst for the conversion of sugars to lactic acid.

**Figure 3:**
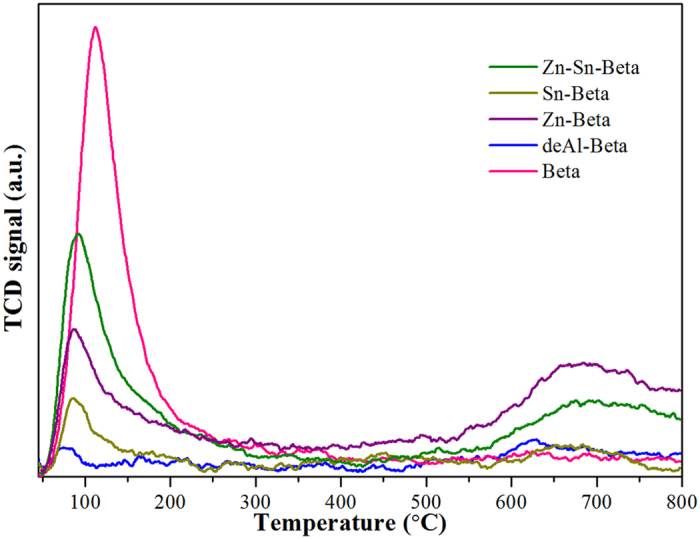
CO_2_-TPD profiles of the different Beta zeolites.

Based on the good performance of the Zn-Sn-Beta zeolite to catalyze sugars to lactic acid in water without an alkali, this catalyst may inhibit the formation of other products, such as 5-HMF and its subsequent decomposition due to the introduction of Zn into the Sn-Beta zeolite, which enhances the strength of base sites besides Lewis acid sites. To confirm this hypothesis, we measured the base properties of these Beta zeolites using CO_2_-TPD experiments (Fig. 3). The base sites of different Beta zeolites were distributed in two desorption regions located at approximately 100 °C and 700 °C, which were associated with moderate and strong base sites, respectively^30^. The rude Beta zeolite possessed the most moderate base sites and no strong base sites. However, the deAl-Beta zeolite has neither moderate nor strong base sites, which suggests that the high Al content of the rude Beta zeolite most likely results in highest quantity of moderate base sites. After the introduction of metals to the deAl-Beta zeolite, the Zn-Sn-Beta zeolite has more moderate base sites than the Sn- or Zn-Beta zeolite due to the possible coupling effect of the two metals. Nevertheless, the Zn-Sn-Beta zeolite has fewer strong base sites than the Zn-Beta zeolite due to the few strong base sites on the Sn-Beta zeolite. To further elucidate the integrated base sites of different Beta zeolites, we investigated the potentiometric titration of the basic sites using HCl solution (Fig. S7). In comparison to Beta, deAl-, and Sn-Beta zeolites, the Zn- and Zn-Sn-Beta zeolites have higher initial pH values (pH = 6.0) and require approximately 10.0 ml of HCl to the reach of titration end point (pH = 2.5). The results from both the CO_2_-TPD and potentiometric titration experiments imply that introduction of Zn can create and enhance the strength of base sites. This result indicates that the Zn-Sn-Beta zeolite primarily consists of strong Lewis acid sites along with a certain amount of base sites. In addition, the combination of acid and base sites may perfectly match the proposed requirements for an ideal catalyst for the conversion of sugars to lactic acid rather than HMF, which would explain the excellent catalytic results obtained with this material in water without an alkali.

### Reaction behavior of intermediates and inhibition of side-product formation

Herein, we report that the yield of lactic acid was as high as 48% when the Zn-Sn-Beta zeolite was employed for the glucose transformation. A cascade reaction must be controlled to produce lactic acid from glucose as follows: the isomerization of glucose to fructose, a retro-aldol reaction of fructose to form two trioses (glyceraldehyde and dioxyacetone), the dehydration of trioses to pyruvic aldehyde and the hydration of pyruvic aldehyde followed by the conversion to lactic acid via 1,2-hydride shift^3^. To further explore the role of the Zn-Sn-Beta zeolite, we investigated the catalytic effects of different Beta zeolites on the production of lactic acid from two key intermediates (i.e., dihydroxyacetone and pyruvaldehyde) in water without a base. As shown in Table S4, the yields for lactic acid from dihydroxyacetone and pyruvaldehyde were 75% and 65%, respectively, over Sn-Beta and 67% and 68%, respectively, over the Zn-Sn-Beta zeolite. However, the yields of lactic acid from dihydroxyacetone and pyruvaldehyde were only 7% and 11% over Zn-Beta. This observation provides evidence that the Zn introduced into the Zn-Sn-Beta zeolite does not increase the yield of lactic acid directly, but may inhibit a series of side reactions related to fructose dehydration to HMF and its subsequent decomposition.

To confirm this hypothesis, we performed the conversion of fructose and HMF with or without 0.125 M lactic acid over different Beta zeolites. To obtain a high yield of lactic acid, the dehydration of fructose to form HMF must be inhibited. This reaction could be enhanced under the action of Brønsted acids from lactic acid in itself. Here, we focused on HMF and its derivatives because the other side-products (i.e., formic acid, acetic acid, levulinic acid and acetol) were formed in small quantities. As expected, the yields of HMF and its derivatives were relatively lower in the presence of Zn. In particular, for the Zn-Sn-Beta zeolite, the yield of HMF and its derivatives was less than 10%, and the highest yield of lactic acid (52%) was produced (Table S5, entry 3). It is important to note that the highest yields of HMF (44.1%) and HMF derivatives (15.4%) were formed in the absence of any catalyst (Table S5, entry 4), which is completely different from glucose under the same conditions (Table 1, entry 1). This result is due to the dehydration of fructose as well as the isomerization reaction from glucose to fructose being limited by the Lewis acid catalyst. When lactic acid was added as a typical Brønsted acid with fructose at the beginning of the reaction, the yields of lactic acid decreased, and HMF and its derivatives increased in all of the experimental trials (Table S5, entries 5–8). Using Sn-Beta as the catalyst, the yield of lactic acid decreased from 27% to 16%, and the yields of HMF and its derivatives increased from 16.2% and 7.9% to 22.8% and 15.3%, respectively (Table S5, entries 1 and 5). However, this trend was weaker in the presence of Zn (Table S5, entries 2, 3, 6 and 7), which may be due to the base sites derived from Zn combine with the Brønsted acid around the catalyst to decrease the formation of HMF and its derivatives. Further experimental studies are needed to clarify the actual mechanism of the base sites in the side reaction of fructose. In addition to the dehydration of fructose, the decomposition of HMF is also detrimental to the production of lactic acid based on the chemical equilibrium. Investigations of the decomposition of HMF were performed. The main products included formic acid, levulinic acid and 2,5-furandicarboxaldegyde. In comparison to the 84% decomposition rate of HMF by Sn-Beta, a much lower decomposition rate was observed for Zn-Beta (47%) and Zn-Sn-Beta (49%) (Table S6, entries 1–3). The introduction of Zn endows the catalysts with base sites, which reduces the conversion of HMF. The presence of lactic acid (0.125 M) in the initial reaction solution lowers the pH of the medium, enhancing the decomposition rate of HMF (Table S6, entries 5–8). However, the conversion of HMF is less over catalysts containing Zn. These results indicate that the base sites in the catalyst have the ability to inhibit the conversion of HMF. Based on these results, in water without a base, the Zn-Sn-Beta with the incorporation of Zn enhanced both the Lewis acid and base sites, and the base sites inhibited the conversion of fructose to HMF, resulting in a high lactic acid yield.

## Conclusion

**Figure 4:**
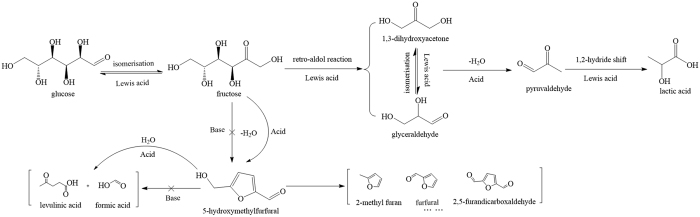
Proposed reaction pathway for the formation of different products from glucose over the Zn-Sn-Beta zeolite.

Based on this discussion, the high activity of the dual metal Zn-Sn-Beta zeolite system has been illustrated in Fig. 4. The introduction of Zn enhances the Lewis acid and creates base sites, and the base sites inhibit the effect of inevitable action of the Brønsted acid from lactic acid to control the dehydration of fructose to HMF and its derivatives. The current results should inspire studies on the development of the conversion of alternative renewable high-valued chemicals to fossil fuels from biomass derivatives via novel zeolites with Lewis acid and base sites.

## Methods

### List of Chemicals

D-(+)-glucose (99.5%), D-(−)-fructose (99%), oxalic acid (99.0%) and tin(II) acetate were obtained from Sigma-Aldrich. Formic acid (≥98%), acetic acid (99.8%), glycolic acid (98%), levulinic acid (99%), pyruvaldehyde (40%) and 1,3-dihydroxyacetone dimer (97%) were purchased from Aladdin. Pyruvic acid (>97%) was purchased from Wako. Lactic acid (1.0 M) was purchased from Alfa Aesar. 5-Hydroxymethylfurfural (98%) was purchased from J&K Scientific. Methyl DL-lactate (>98%) and zinc acetate were purchased from Sinopharm Chemical Reagent Co., Ltd.

### Catalyst synthesis

A commercial Beta zeolite (Catalyst Plant of Nankai University) with a Si/Al_2_ ratio of 25 was dealuminated by treatment with a HNO_3_ solution (65%, w/w) at 80 °C for 20 h. Then, the white precipitates were collected by centrifugation and followed by repeated washing with deionized water until the supernatant was neutral. Then, the obtained dealuminated Beta zeolite (deAl-Beta) was dried at 150 °C overnight. Solid-state ion exchange was performed by grinding the deAl-Beta, tin (II) acetate and zinc (II) acetate for 30 min. Finally, the samples were calcined in static air at 550 °C with a ramp of 2 °C per minute for 6 h^19^.

### Catalyst characterization

The powder X-ray diffraction (XRD) measurements were performed on a Bruker D8 Advance X-ray powder diffractometer (40 kV, 40 mA, CuK radiation of = 1.54 Å) over a 2θ range of 5–70 °C at room temperature. The Raman spectrum were recorded at room temperature on a Horiba Jobin Yvon XploRA Raman spectrometer using a 50 mW laser source at an excitation wavelength of 532 nm. The spectral resolution was 1.8 cm^−1^. The nitrogen adsorption-desorption data were obtained using a Micromeritics ASAP2020M analyzer at −196 °C. Prior to the measurement, the sample was degassed at 150 °C for 6 h in the vacuum line. The specific surface area was calculated by employing the Brunauer-Emmett-Teller (BET) method in a range of relative pressures from 0.005 to 0.25. The pore-size distribution was derived from the desorption branches of the isotherms using the Barrett-Joyner-Halenda (BJH) method. The Si, Al, Sn, and Zn contents in the catalysts were determined by inductively coupled plasma optical emission spectroscopy (ICP-OES, Perkin Elmer Optima 2100 DV). Prior to the measurements, the samples were digested in an acidic mixture (HCl-HNO_3_-HF) at 150 °C for 12 h. The acidic properties of the catalysts were studied by adsorption and temperature programmed desorption (TPD) of pyridine using Fourier transform infrared (FT-IR) spectroscopy. The infrared spectra were recorded on a Perkin Elmer Frontier FT-IR from 1400 to 1700 cm^−1^ with a resolution of 2 cm^−1^. A 10 mg of sample was pressed into a self-supported wafer with a diameter of 13 mm. The wafer was placed in a quartz IR cell, which was sealed with CaF_2_ windows and connected to a vacuum system. The samples were dried at 450 °C for 2 h under vacuum. After cooling to RT, pyridine vapor was admitted into the cell, and the adsorption lasted for 0.5 h. Then, the desorption steps at 150 °C (1 h) and, 250 °C (1 h) were performed. Potentiometric titration of the basic sites was conducted with an automatic titrator (907 Titrando, Metrohm, Switzerland). The sample (200 mg) was suspended in 50 ml of KCl (0.0010 M) followed by sonication for 10 min. A volumetric standard HCl solution (0.0245 M) was used as the titrant, starting from the initial pH of the catalyst suspension. The measurements of the basicity of samples were also performed on Micromeritics AutochemII 2920 chemisorption analyzer using the CO_2_-TPD method. The sample was purged with Ar and heated to 550 °C at a rate of 10 °C per minute, which was maintained for 1 h and then cooled to 45 °C. The surface of the catalyst was saturated with CO_2_ for 30 min at this temperature, and then, the excess CO_2_ was purged with Ar at 45 °C. The TPD curve of the sample was measured by increasing the temperature from 45 to 800 °C at a heating rate of 10 °C /min under Ar at a flow rate of 30 cm^3^/min.

### Catalytic reactions

The catalytic reactions were performed in a closed Teflon vessel (50 ml) in a stainless steel autoclave. In a typical procedure, the vessel was charged with carbohydrate (7.5 mmol carbon), catalyst (160 mg), and solvent (10 g) followed by heating to the desired reaction temperature (463 K) in a rotating oven (20 rpm). After 2 hours of stirring, the autoclave was cooled, and the reaction mixture was analyzed. All of the experiments were replicated at least three times, and the mean values are reported. In the reuse experiments, after each test, the catalyst was recovered and calcined at 550 °C for 6 h in static air.

### Products analysis

The concentration of carbohydrates was analyzed on an Agilent 1200 series HPLC (Shodex SUGAR SH1011) with a RI detector using a 0.0050 M aqueous sulphuric acid solution as the eluent at a flow rate of 0.8 ml/min. The yields of organic acids (i.e., lactic acid, glycolic acid, formic acid, acetic acid, oxalic acid and levulinic acid) and 5-hydroxymethyfurfural were determined by HPLC (Gemini-NX 5u C18) with a UV detector (210 nm) using a 0.1% aqueous phosphoric acid as the eluent at a flow rate of 1 ml/min based on the external standard. To confirm the products, a Shimadzu QP2010 GC-MS equipped with a HP-5ms was employed. Prior to the analyses, the reaction mixture (0.5 ml) was stabilized with NaOH (1 M), and the water in the mixture was removed by freeze drying. The crude product was thoroughly dissolved in dimethylsulphoxide (0.5 ml) and then mixed with a silylating agent (bis-(trimethylsilyl) trifluoroacetamide +1% trimethylchlorosilane (1 ml, Regis Technologies, Inc. USA). This solution was maintained at 65 °C for 2 h to ensure the complete silylation and subsequently injected into the GC-MS (Fig. S8a). The derivatives of 5-hydroxymethyfurfural are not commercially available. The derivatives were identified by GC-MS, and the yields were calculated based on calibration data for 5-hydroxymethyfurfural by assuming similar carbon response factors. Prior to measurement of these derivatives, the reaction mixture was extracted using dichloromethane three times followed by analysis on an Agilent 7890A GC system coupled with an Agilent 5975C mass detector and an HP-INNOWAX capillary column (Fig. S8b). The reaction mixture with methanol as the solvent was analyzed on a GC (Agilent 7820A instrument) equipped with a DB-WAXetr capillary column and an FID detector for qualitative analysis. In addition, lactic acid was identified by matching both the 1H-NMR (Fig. S9) and the HPLC retention time of the lactic acid standard sample. The conversion of sugars and the product yields were determined based on carbon. The total organic carbon (TOC) measurement was determined using an Analytikjena Multi N/C 2100 analyzer.

## Additional Information

**How to cite this article**: Dong, W. *et al.* Selective Chemical Conversion of Sugars in Aqueous Solutions without Alkali to Lactic Acid Over a Zn-Sn-Beta Lewis Acid-Base Catalyst. *Sci. Rep.* **6**, 26713; doi: 10.1038/srep26713 (2016).

## Supplementary Material

Supplementary Information

## Figures and Tables

**Table 1:** Conversion of glucose using different Beta zeolites.

Entry	Catalyst	Conversion (%)	Yield (%)						
			Lactic acid	Formic acid	Acetic acid	Levulinic acid	Acetol	HMF	HMF derivatives
1	No catalyst	21	4	3.6	0.2	3.0	0	3.3	1.2
2	Beta	78	5	2.1	0.6	3.3	0.1	14.6	7.5
3	de-Al Beta	37	3	1.8	0.1	2.4	1.0	7.1	3.7
4	Sn-Beta	97	23	0.7	1.4	0.5	2.2	17.3	12.1
5	Zn-Beta	95	17	2.3	1.2	2.8	2.8	12.2	7.3
6	Zn-Sn-Beta	>99	48	2.0	1.8	5.2	1.3	7.9	3.9
7	SnO_2_^a^	92	6	2.2	0.7	1.9	0.4	5.9	2.3
8	ZnO^a^	85	12	3.6	0.9	3.4	2.1	14.1	6.4

Glucose (225 mg), catalyst (160 mg), and water (10 ml) were stirred in an autoclave at 190 °C for 2 hours. The yield is calculated on a carbon basis and reported as mean values.

^a^Equivalent molar amount of Sn or Zn as for 160 mg of Zn-Sn-Beta.
