# Supplementary material for: Selective Chemical Conversion of Sugars in Aqueous Solutions without Alkali to Lactic Acid Over a Zn-Sn-Beta Lewis Acid-Base Catalyst
Source: Sci Rep. 2016 May 25;6:26713. doi: 10.1038/srep26713 (PMC4879548; doi:10.1038/srep26713)
Supplement: Supplementary Information [file srep26713-s1.doc]

**SUPPORTING INFORMATION**

Selective Chemical Conversion of Sugars in Aqueous Solutions without Alkali to Lactic Acid Over a Zn-Sn-Beta Lewis Acid-Base Catalyst

Wenjie Dong^1^, Zheng Shen^1,*^, Boyu Peng^1^, Minyan Gu^1^, Xuefei Zhou^1^, Bo Xiang^2^, and Yalei Zhang^1,*^

^1^State Key Laboratory of Pollution Control and Resources Reuse, National Engineering Research Center of Facilities Agriculture, Key Laboratory of Yangtze River Water Environment of Ministry of Education, College of Environmental Science and Engineering, Tongji University, Shanghai 200092, China

^2^Department of Chemistry, Tongji University, Shanghai 200092, China

*Correspondence to: shenzheng78@163.com, zhangyalei2003@163.com

Figures S1-S9

Tables S1-S6

**Supporting Figures**


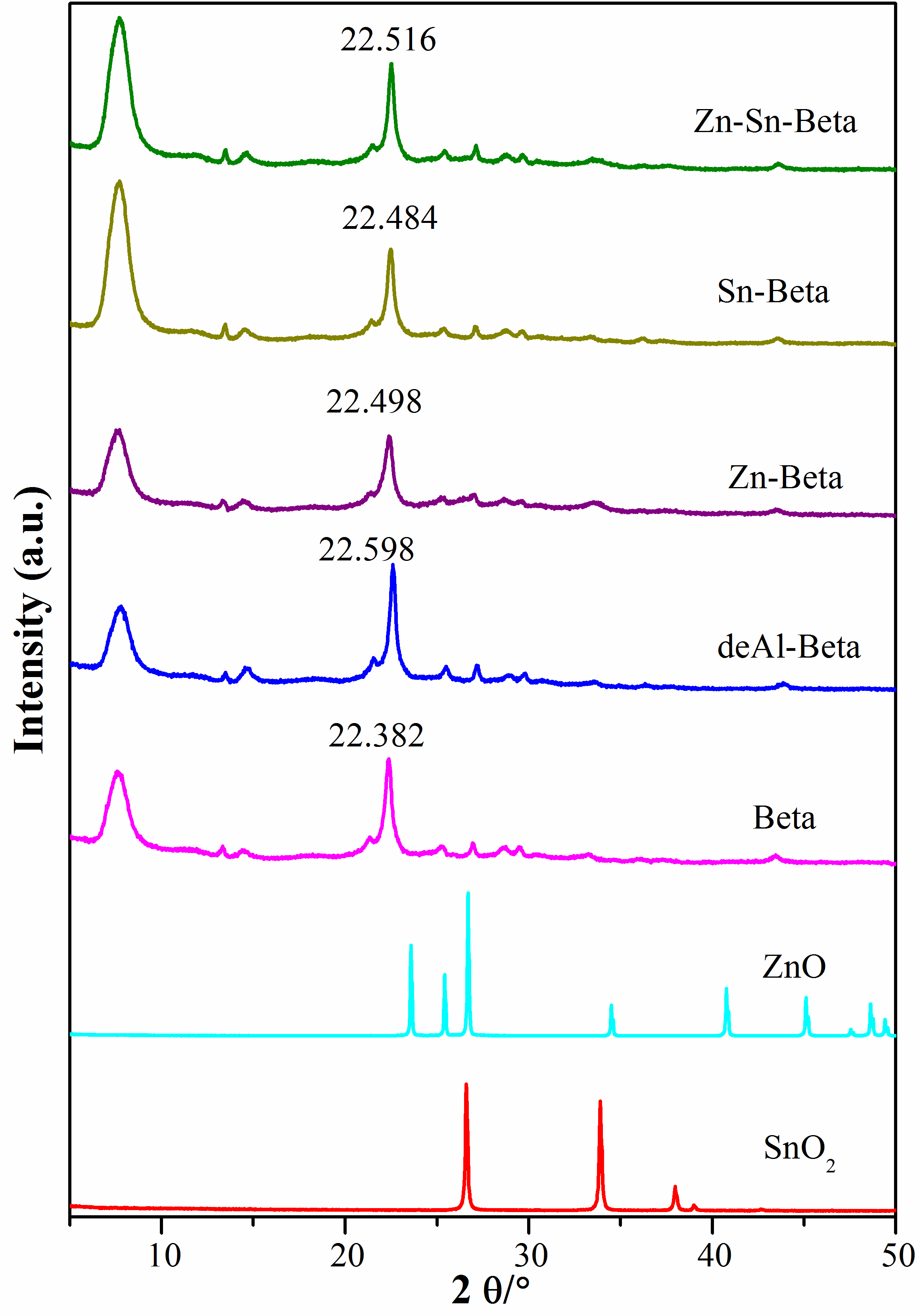


**Figure S1.** XRD of SnO_2_, ZnO, and different Beta zeolites.

**
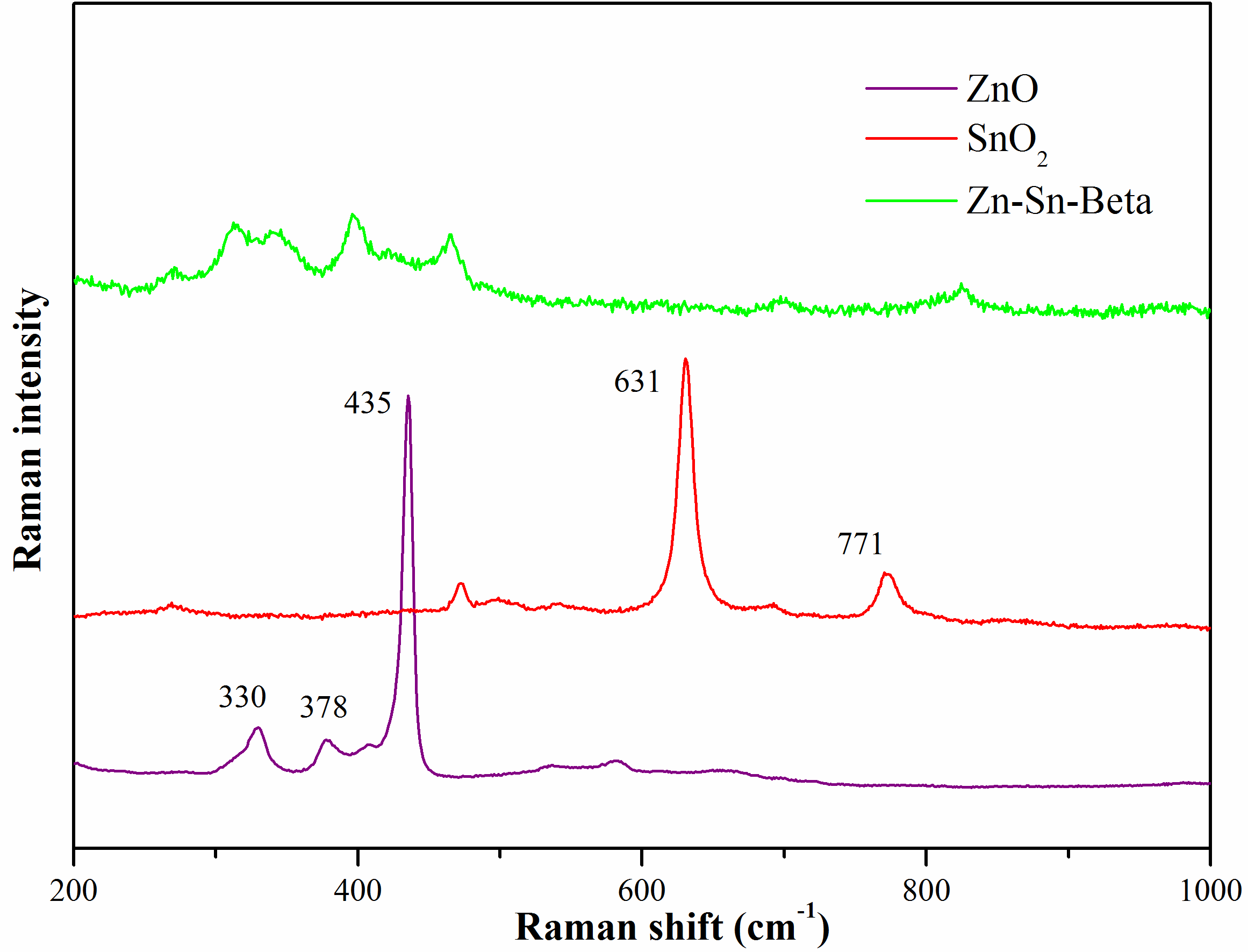
**

**Figure S2.** Raman spectra of ZnO, SnO_2_ and Zn-Sn-Beta zeolite.


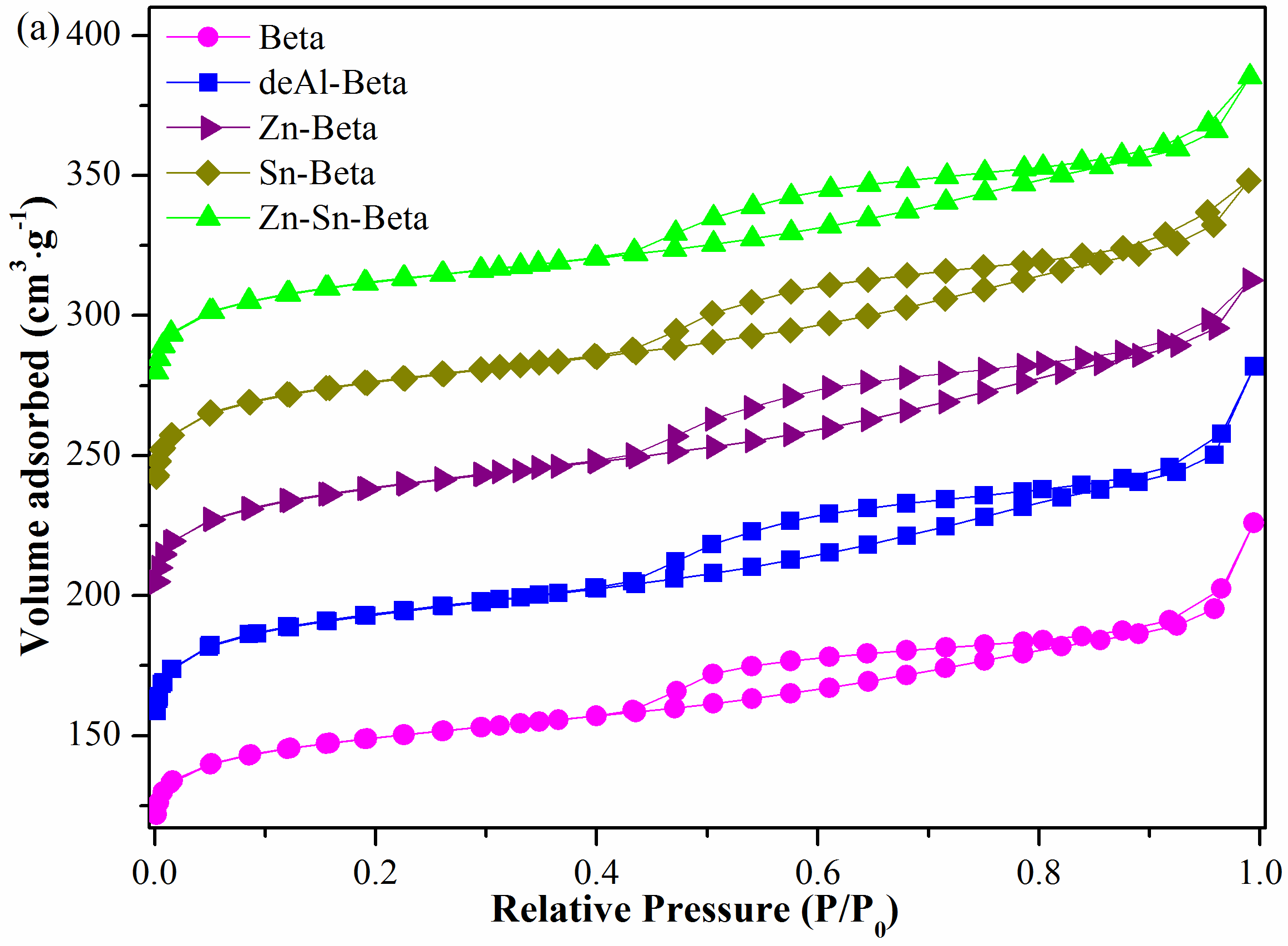


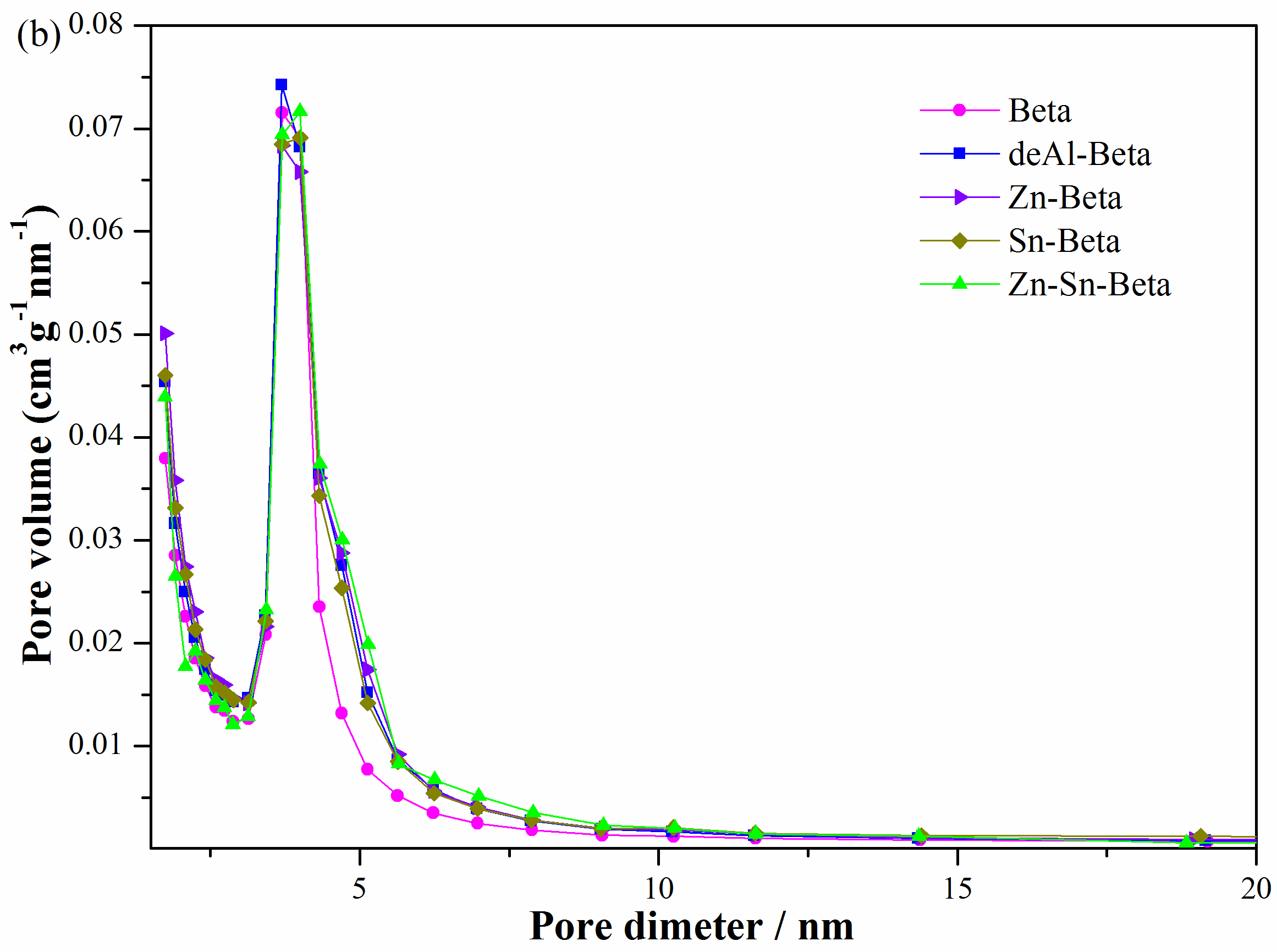


**Figure S3.** (a) Nitrogen adsorption/desorption isotherms of different Beta zeolites. Individual samples have been moved in steps of 40 for illustrative purpose. (b) corresponding distributions of pore diameters obtained from the desorption branch using the BJH method.

.


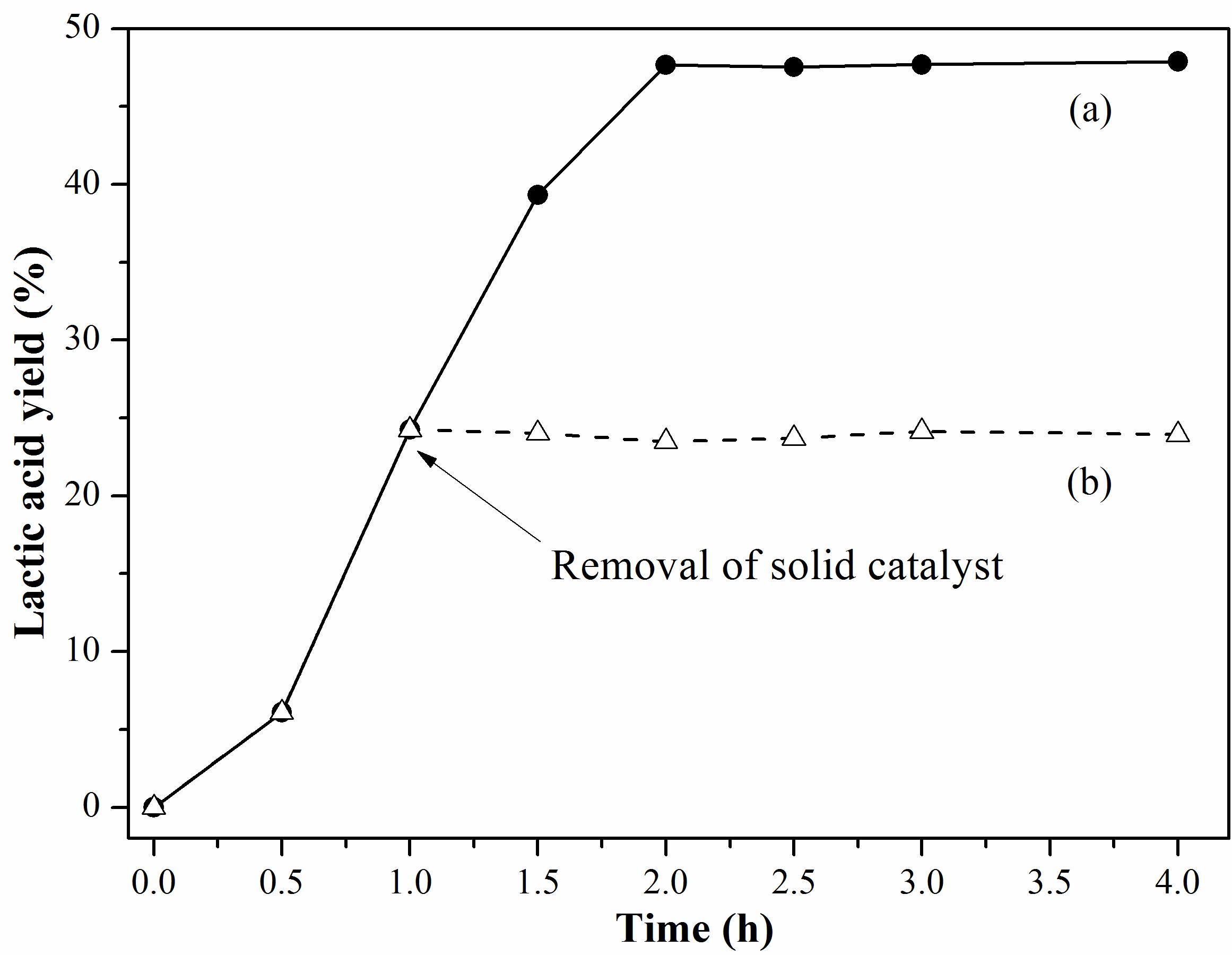


**Figure S4.** (a) Time-dependent yield of lactic acid on the conversion of glucose over Zn-Sn-Beta zeolite, and (b) hot-filtration test for the catalyst (225 mg glucose, 10 ml water, 160 mg catalyst, 190°C)


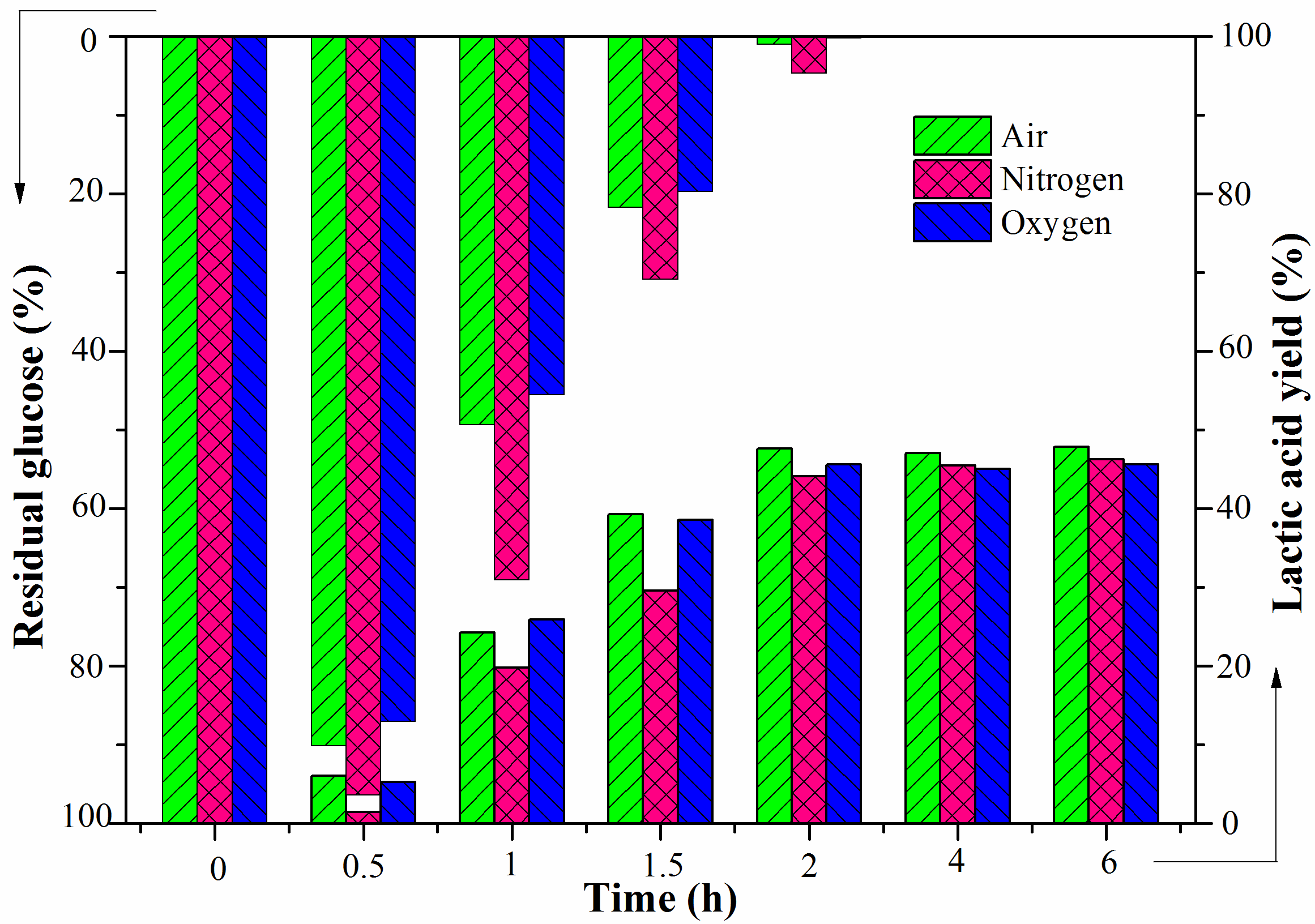


**Figure S5.** Effect of gas environment on the conversion of glucose to lactic acid with Zn-Sn-Beta zeolite (225 mg glucose, 10 ml water, 160 mg catalyst, 190°C).


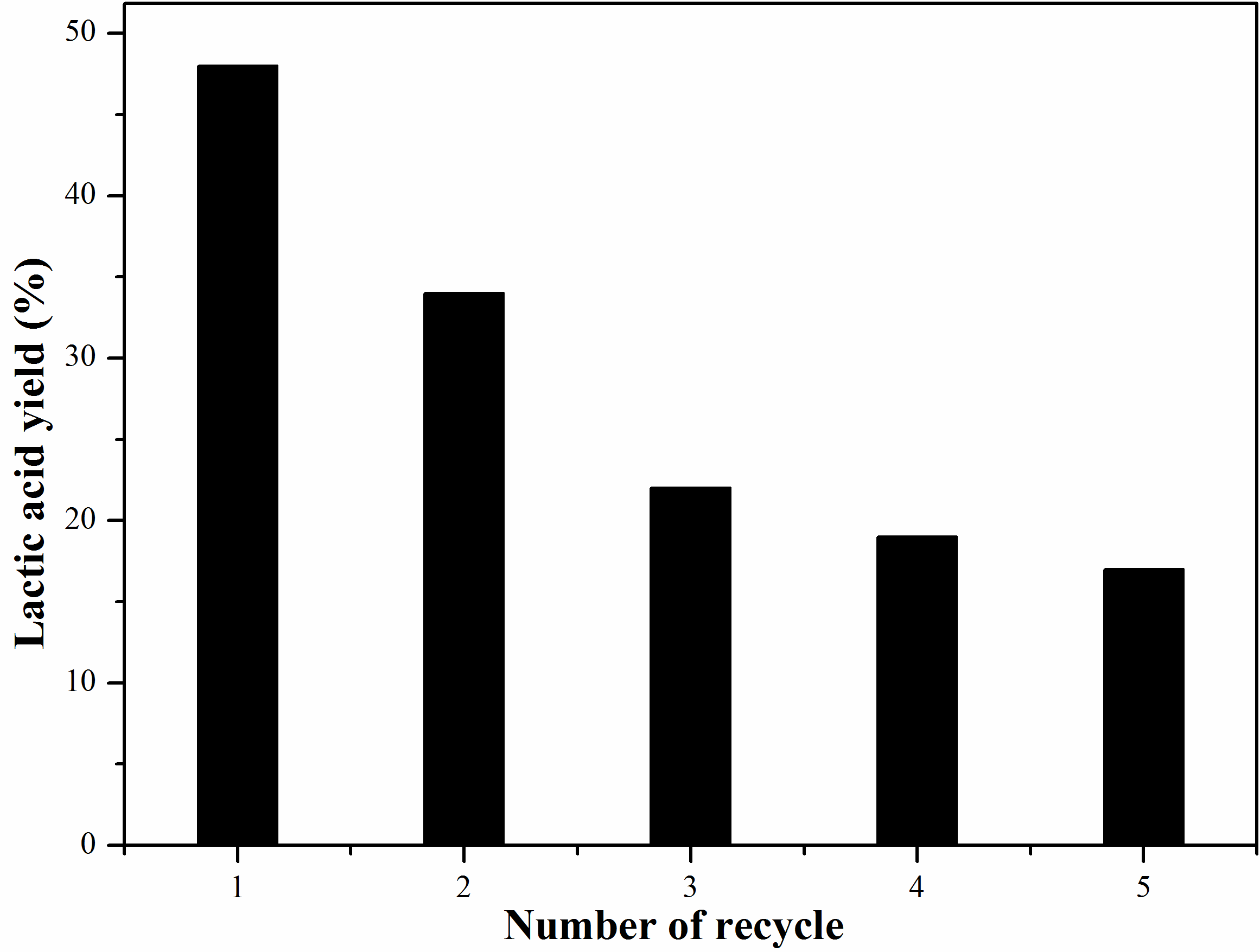


**Figure S6.** Recycling test of Zn-Sn-Beta zeolite for the conversion of glucose (225 mg glucose, 10 ml water, 160 mg catalyst, 190°C)


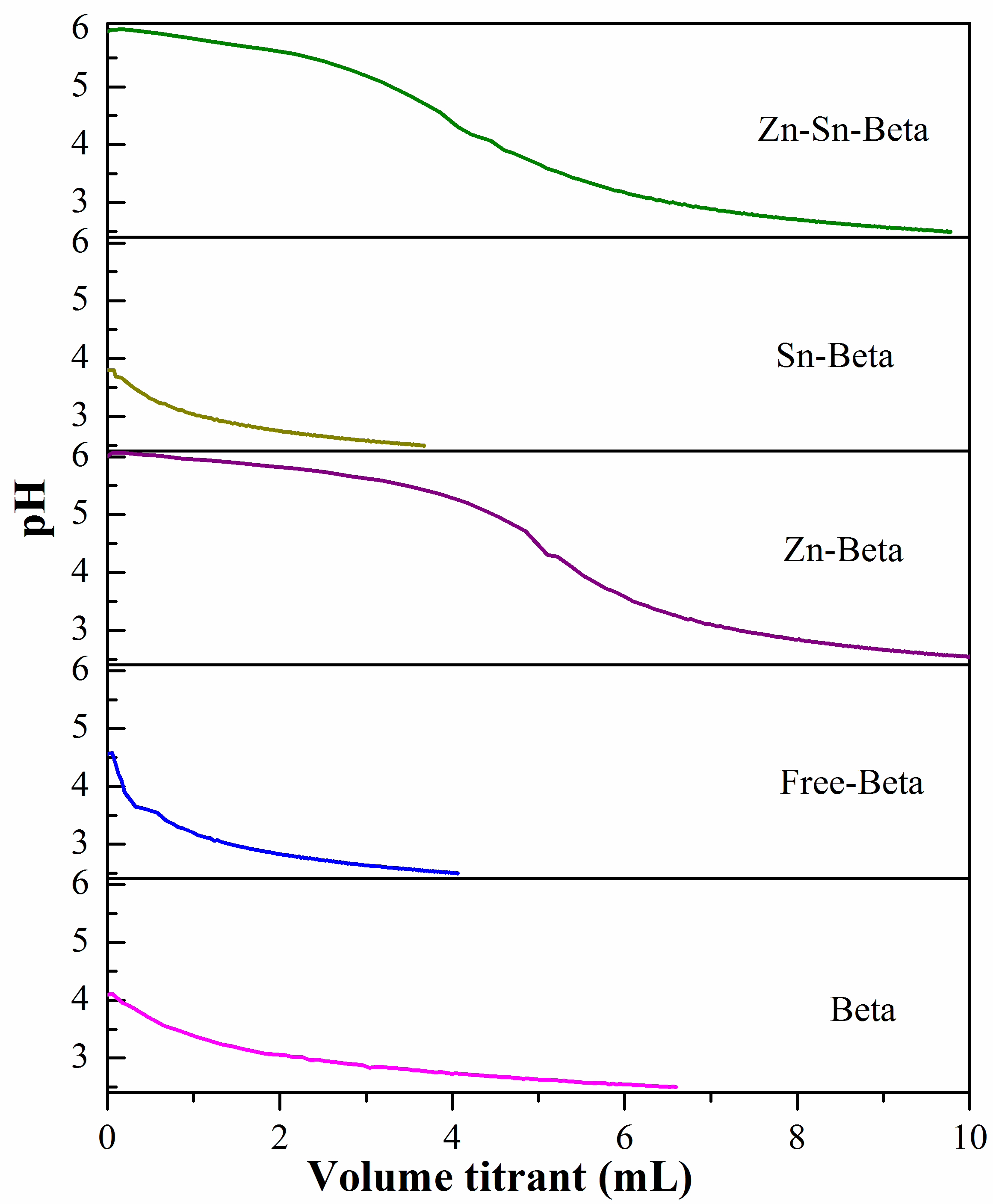


**Figure S7.** HCl titration curves of different Beta zeolites.


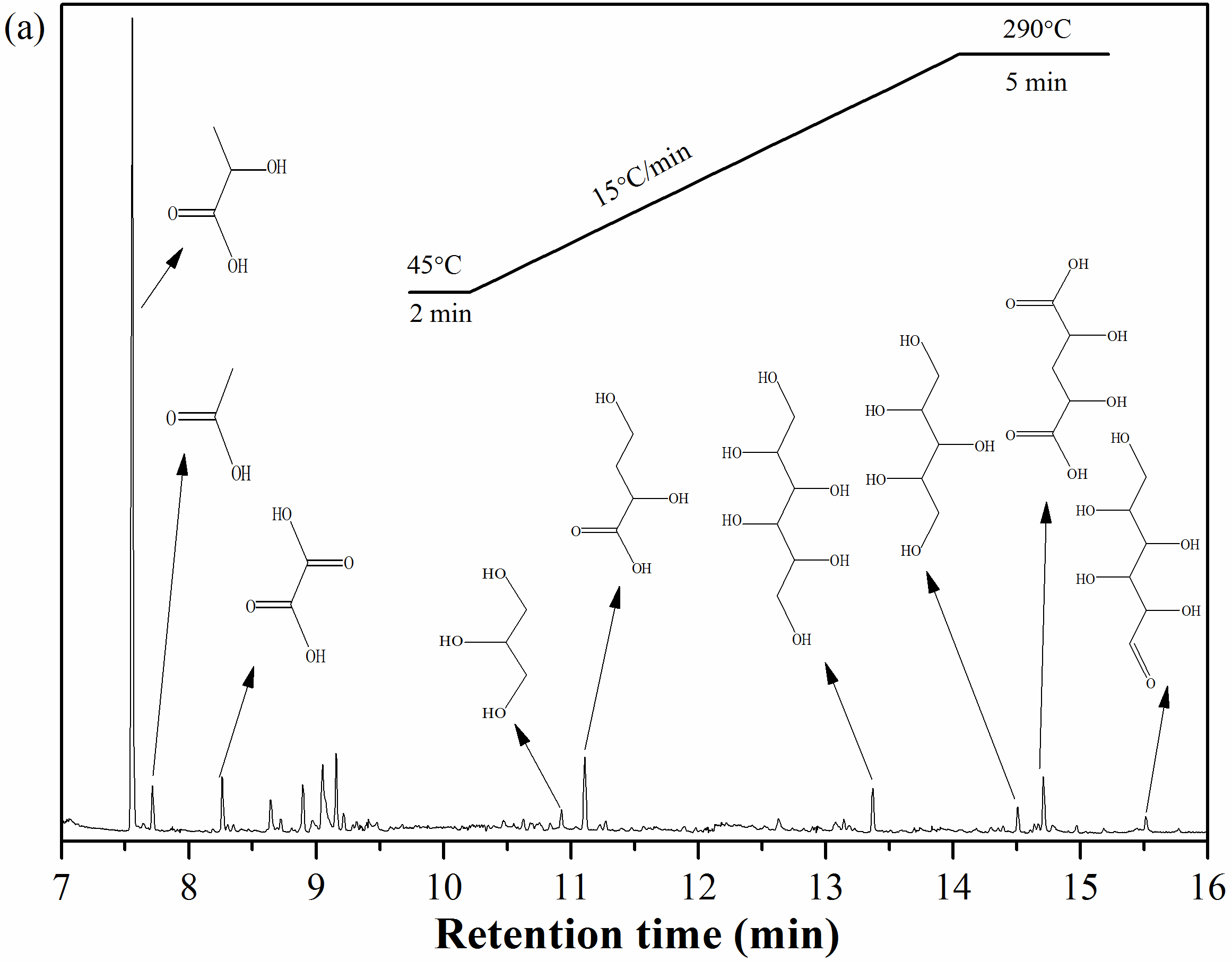


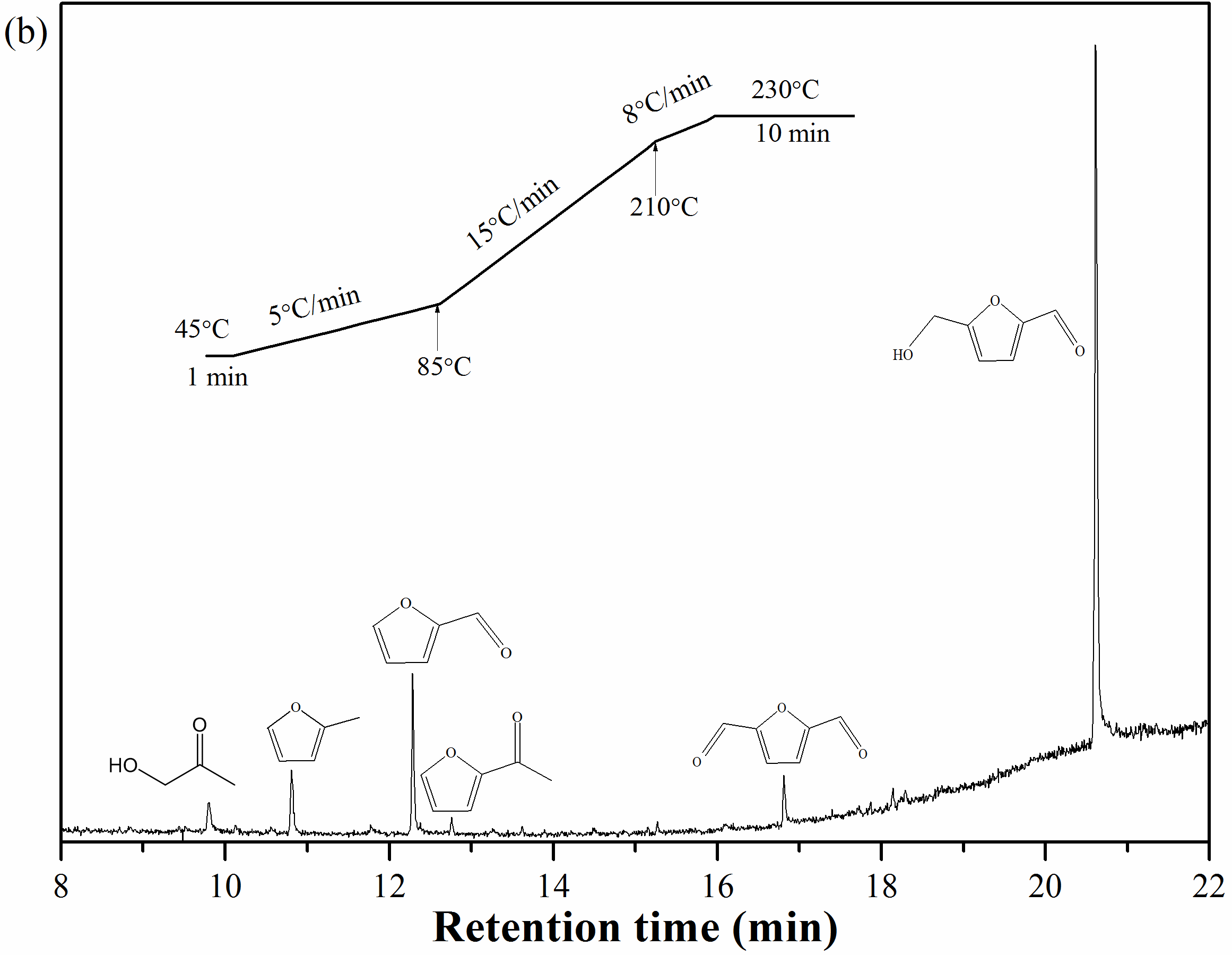


**Figure S8.** GC-MS chromatogram of sample obtained after the conversion of glucose over Zn-Sn-Beta zeolite at 190°C for 2 h. (a) GC-MS chromatogram of silylated sample, and (b) GC-MS chromatogram of sample through extraction with dichloromethane.


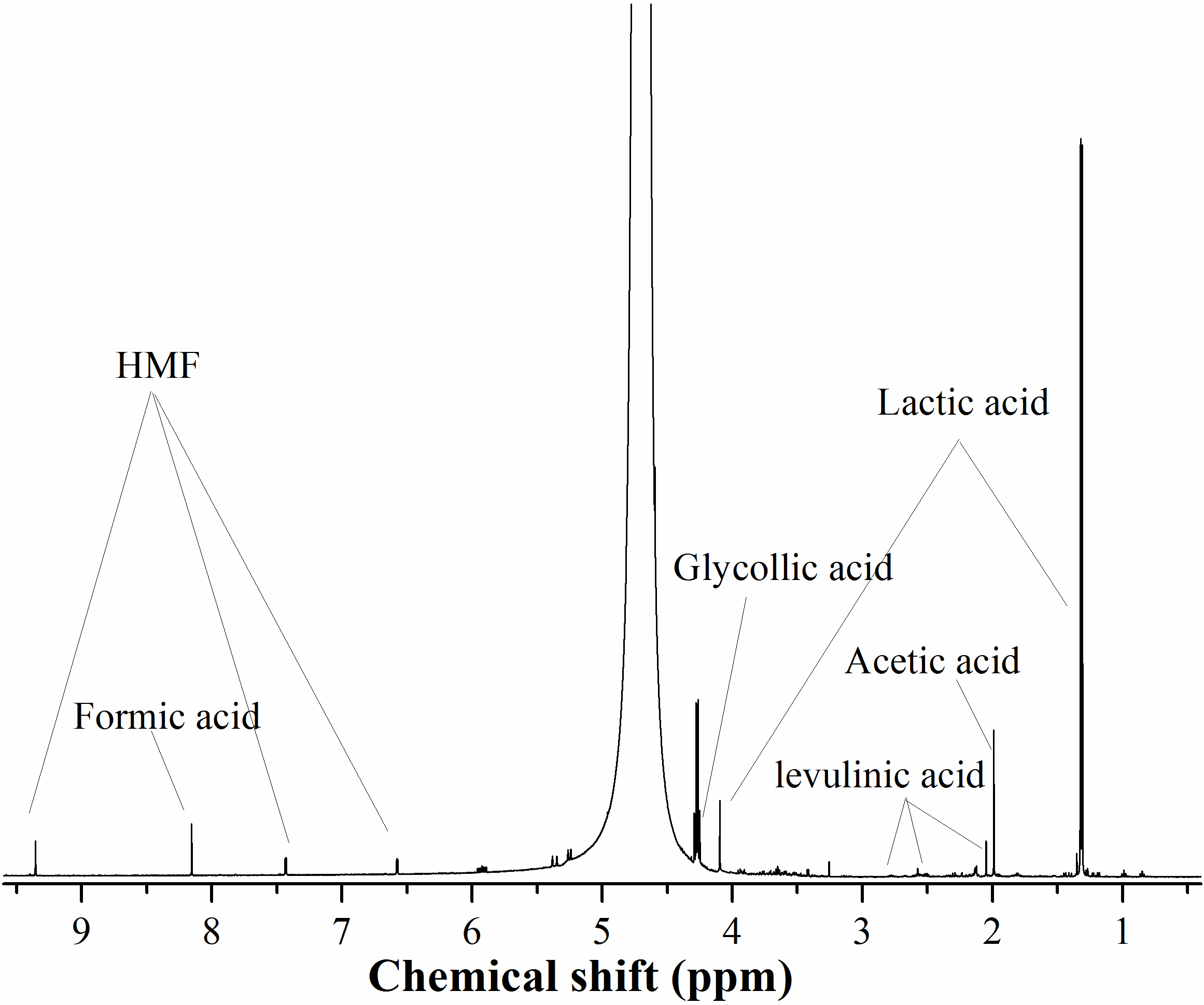


**Figure S9.** ^1^H NMR spectrum of sample obtained after the conversion of glucose over Zn-Sn-Beta zeolite at 190°C for 2 h.

**Supporting Tables**

**Table S1.** Physicochemical properties of different Beta zeolites.

| Entry | Catalyst | S_BET_^a^ | V_total_^b^ | V_micropore_^c^ | Sn^d^ | Zn^d^ | Si/Al ratio^c^ |
| --- | --- | --- | --- | --- | --- | --- | --- |
|  |  | (m^2^g^-1^) | (mlg^-1^) | (mlg^-1^) | (mmolg^-1^) | (mmolg^-1^) |  |
| 1 | Beta | 581 | 0.350 | 0.186 | - | - | 25 |
| 2 | deAl-Beta | 582 | 0.374 | 0.184 | - | - | >1700 |
| 3 | Zn-Beta | 610 | 0.359 | 0.191 | - | 0.34 | >1700 |
| 4 | Sn-Beta | 603 | 0.353 | 0.189 | 0.35 | - | >1700 |
| 5 | Zn-Sn-Beta | 587 | 0.348 | 0.185 | 0.34 | 0.34 | >1700 |

^a^Brunauer-Emmet-Teller surface area calculated by N_2_ physisorption; ^b^Evaluated at P/P_0_ = 0.99; ^c^Determined using the t-plot method; ^d^Determined by ICP-OES analysis.

**Table S2.** The lactic acid or methyl lactate yield obtained using Zn-Sn-Beta zeolite with various sugars^a^.

| Entry | Substrate | Conversion (%) | Lactic acid yield (%) | Methyl lactate yield (%) |
| --- | --- | --- | --- | --- |
| 1 | Glucose | >99 | 48 | 30 |
| 2^b^ | Glucose | >99 | 47 | 27 |
| 3 | Fructose | >99 | 52 | 29 |
| 4 | Mannose | >99 | 45 | 28 |
| 5 | Galactose | >99 | 47 | 25 |
| 6 | Sucrose | >99 | 54 | 32 |
| 7 | Lactose | >99 | 35 | 6 |
| 8 | Cellose | >99 | 31 | 4 |

^a^ sugars (7.5 mmol C), 10 ml water, 160 mg catalyst, 190°C, 2 h.

^b^ sugars (7.5 mmol C), 10 ml methanol, 160 mg catalyst, 160°C, 20 h.

**Table S3.** Acid types and quantity of different Beta zeolites.

| Temperature | 150 | | 250 | | 350 | | 450 | |
| --- | --- | --- | --- | --- | --- | --- | --- | --- |
| Catalysts | B | L | B | L | B | L | B | L |
|  | mmolg^-1^ | | | | | | | |
| Beta | 0.17 | 0.15 | 0.17 | 0.13 | 0.12 | 0.11 | 0.07 | 0.11 |
| de-Al Beta | 0 | 0 | 0 | 0 | 0 | 0 | 0 | 0 |
| Zn-Beta | 0 | 0.12 | 0 | 0.07 | 0 | 0.04 | 0 | 0.03 |
| Sn-Beta | 0.03 | 0.13 | 0 | 0.04 | 0 | 0 | 0 | 0 |
| Zn-Sn-Beta | 0.01 | 0.17 | 0.01 | 0.10 | 0 | 0.05 | 0 | 0.02 |

**Table S4.** Yield of lactic acid from dihydroxyacetone and pyruvaldehyde over different catalysts.

| Entry | Catalyst | Dihydroxyacetone | | Pyruvaldehyde | |
| --- | --- | --- | --- | --- | --- |
|  |  | Conversion (%) | Yield (%)  Lactic acid yield (%) | Conversion (%) | Yield (%)  Lactic acid yield (%) |
| 1 | Sn-Beta | >99 | 75 | >99 | 65 |
| 2 | Zn-Beta | >99 | 7 | 92 | 11 |
| 3 | Zn-Sn-Beta | >99 | 67 | >99 | 68 |
| 4 | No catalyst | >99 | 2 | 29 | 2 |

Entry 1-4: 10 ml dihydroxyacetone or pyruvaldehyde solution (0.125 M), 80 mg catalyst, 190°C, 2h.

**Table S5.** Effect of lactic acid for fructose conversion over different catalysts.

| Entry | Catalyst | Conversion | Yield (%) | | | | | | |
| --- | --- | --- | --- | --- | --- | --- | --- | --- | --- |
|  |  | (%) | Lactic acid | Formic acid | Acetic acid | Levulinic acid | Acetol | HMF | HMF derivatives |
| 1 | Sn-Beta | 99 | 27 | 1.1 | 1.3 | 1.3 | 2.5 | 16.2 | 7.9 |
| 2 | Zn-Beta | 98 | 18 | 1.9 | 0.9 | 1.6 | 3.2 | 11.4 | 4.6 |
| 3 | Zn-Sn-Beta | >99 | 52 | 1.0 | 1.5 | 3.4 | 0.4 | 6.3 | 3.5 |
| 4 | No catalyst | 91 | 3 | 0.9 | 0.3 | 5.6 | 0.1 | 44.1 | 15.4 |
| 5 | Sn-Beta | >99 | 16 | 1.9 | 0.2 | 2.1 | 0.8 | 22.8 | 15.3 |
| 6 | Zn-Beta | >99 | 12 | 1.4 | 0.4 | 6.5 | 3.9 | 14.1 | 11.3 |
| 7 | Zn-Sn-Beta | >99 | 31 | 2.4 | 0.9 | 3.4 | 0.4 | 12.2 | 8.7 |
| 8 | No catalyst | >99 | 2 | 2.1 | 0.3 | 1.7 | 0.2 | 52.4 | 20.3 |

Entry 1-4: 225 mg fructose, 10 ml water, 160 mg catalyst, 190°C, 2h;

Entry 5-8: 225 mg fructose, 10 ml lactic acid solution (0.125 M), 160 mg catalyst, 190°C, 2h.

**Table S6.** Effect of lactic acid for 5-hydroxymethyfurfural conversion over different catalysts.

| Entry | | Catalyst | Conversion | Yield (%) | | |
| --- | --- | --- | --- | --- | --- | --- |
|  |  |  | (%) | Formic acid | levulinic acid | 2,5-furandicarboxaldegyde |
| 1 | Sn-Beta | | 84 | 2.1 | 3.9 | 2.7 |
| 2 | Zn-Beta | | 47 | 1.4 | 0.6 | 5.1 |
| 3 | Zn-Sn-Beta | | 49 | 3.4 | 1.7 | 5.6 |
| 4 | No catalyst | | 59 | 0.8 | 2.5 | 11.5 |
| 5 | Sn-Beta | | 85 | 1.6 | 3.0 | 1.9 |
| 6 | Zn-Beta | | 67 | 0.7 | 3.6 | 3.7 |
| 7 | Zn-Sn-Beta | | 71 | 2.3 | 2.8 | 2.4 |
| 8 | No catalyst | | 81 | 0.6 | 3.5 | 5.4 |

Entry 1-4: 5 ml 5-hydroxymethyfurfural solution (0.0317 M), 5 ml water, 160 mg catalyst, 190°C, 2h;

Entry 5-8: 5 ml 5-hydroxymethyfurfural solution (0.0317 M), 5 ml lactic acid solution (0.25 M), 160 mg catalyst, 190°C, 2h
